# Supplementary material for: Expression Analysis of Outer Membrane Protein HPS_06257 in Different Strains of Glaesserella parasuis and Its Potential Role in Protective Immune Response against HPS_06257-Expressing Strains via Antibody-Dependent Phagocytosis
Source: Vet Sci. 2022 Jul 6;9(7):342. doi: 10.3390/vetsci9070342 (PMC9316402; doi:10.3390/vetsci9070342)
Supplement: Supplementary file 1 [file vetsci-09-00342-s001.zip › vetsci-1763403-supplementary.pdf]

Table S1. Primers used to amplify *HPS_06257* gene

**Primer name**	**Primer sequence (5'-3')**
HPS_06257-Forward (*Bam*H I)	AGT*GGATCC*AATCCGCCCTCTCA
HPS_06257-Reverse (*Xho* I)	TTA*CTCGAG*TGCCAGAAAATCCTTC

Note: 1) these primers were used to amplify *HPS_06257* gene; 2) these primers contained *Bam*H I and *Xho* I sites in the forward and reverse primers, respectively; 3) The restriction enzyme sites were underlined as above.
